# Optimising CO_2_ level and light quality for enhanced whole-cell biotransformation reactions in *Synechocystis* sp. PCC 6803

**DOI:** 10.1186/s12934-025-02828-4

**Published:** 2025-08-31

**Authors:** Michal Hubáček, Lauri Nikkanen, Yagut Allahverdiyeva

**Affiliations:** Molecular Plant Biology Unit, Department of Life Technologies, University of Turku, Turku, Finland

**Keywords:** Cyanobacteria, Biotransformation, Photosynthesis, Lactone, BVMO, Ene-reductase, Biocatalysis

## Abstract

Cyanobacteria are emerging as a promising platform for whole-cell biotransformation, harnessing solar energy to drive biocatalytic reactions through recombinant enzymes. However, optimisation remains challenging due to the complexity of the cyanobacterial metabolism and the regulatory framework in which heterologous enzymes operate. While many enzymes have been deployed for light-driven whole-cell biotransformations, the different experimental conditions used between studies make direct comparison and systematic improvement difficult. We investigated the performance of two Baeyer-Villiger monooxygenases (BVMO) and the ene-reductase YqjM, heterologously expressed in the model cyanobacterium *Synechocystis* sp. PCC 6803, under varying growth and production conditions. NADPH and O_2_ availability, along with protein accumulation levels, were examined as potential bottlenecks affecting enzyme activity. A 4-fold improvement in specific activity of BVMOs was achieved when cultures were grown under elevated CO_2_, and a 2-fold improvement was observed under broad white light enriched with red and blue wavelengths. Elevated CO_2_ cultivations enhanced BVMO protein accumulation, while YqjM levels and activity remained unchanged. In contrast, the modified light spectrum led to a non-significant increase in BVMO accumulation but significantly enhanced specific activity under ambient CO_2_ conditions. These findings demonstrate the importance of a tailored optimisation strategy for each enzyme in cyanobacterial light-driven whole-cell biotransformation and shed light on the complex physiological responses of production strains to environmental conditions.

**Supplementary Information:**

The online version contains supplementary material available at 10.1186/s12934-025-02828-4.

## Background

Cyanobacteria are promising hosts for light-driven whole-cell biotransformation (hereafter biotransformation), an emerging alternative to traditional (bio)catalysis. In these processes, recombinant enzymes expressed in living cells catalyse specific reactions by converting the supplied substrate into a desired product. Many enzymes require cofactors such as NAD(P)H or ATP, which in heterotrophic organisms (bacteria, yeast, fungi, etc.) are regenerated by metabolising externally added sugars to drive biotransformation. However, this typically increases production costs and environmental impact unless waste- or side-stream substrates are used. In contrast, cyanobacteria harness light energy through photosynthesis to continuously regenerate the cofactors required for enzymatic reactions, making light the primarily driver of biotransformation. However, enzymatic activity can persist in the dark if cofactors are provided by the catabolism of storage compounds like glycogen via the oxidative pentose phosphate or Embden-Meyerhof-Parnas pathways.

Several enzymes have been successfully expressed in cyanobacteria, namely monooxygenases performing Baeyer-Villiger oxidation (BVMO), ene-reductases and the cytochrome P450 enzyme family [1, 3, 11, 15, 21, 22, 49]. Among them, the ene-reductase YqjM has shown the highest specific activity in *Synechocystis* sp. PCC 6803 (hereafter *Synechocystis*) reaching up to 166 U g_DCW_^−1^ at OD_750_ = 2 [3]. Several BVMOs from different organisms have been frequently explored, including those from *Parvibaculum lavamentivorans* (Parvi), *Burkholderia xenovorans* (Xeno), *Acinetobacter calcoaceticus* and *Acidovorax* sp., with reported activities ranging from 5 to 63 U g_DCW_^­1^ in different conditions [11, 49, 50]. Among the BVMOs, Xeno demonstrates higher catalytic activity in whole-cell biotransformation compared to Parvi (k_cat−Xeno_ = 103 ± 3 min^­1^, K_M−Xeno_ = 23 ± 5 µM; Erdem et al., [11]. These enzymes are classified as Type-I BVMO, with a strong affinity to NADPH and requirement for flavin adenine dinucleotide (FAD) as a cofactor [26, 30]. In addition to NADPH, BVMOs require molecular oxygen (O_2_) for their catalytic activity, which presents a major limitation when using heterotrophic organisms for biotransformation. Cyanobacteria, on the other hand, are promising hosts due to their innate ability to produce O_2_ through photosynthesis [19, 20].

In oxygenic photosynthesis, light energy drives water oxidation at photosystem II (PSII), releasing electrons, protons and O_2_. The electrons are transported through the photosynthetic electron transport chain (PETC) and ultimately reach ferredoxin (Fd) via photosystem I (PSI). Reduced Fd then donates electrons to ferredoxin-NADP^+^ reductase (FNR), which catalyses the reduction of NADP^+^ to NADPH. Functioning as a central electron hub downstream of PSI, reduced Fd distributes electrons not only to the Calvin-Benson-Bassham (CCB) cycle but also to a variety of pathways, including flavodiiron proteins (FDPs), nitrate assimilation, cyclic electron transport, and the thioredoxin system [14, 35]. *Synechocystis* possesses four FDPs, designated Flv1 to Flv4. FDPs form functional hetero-oligomers (Flv1/3 and Flv2/4) that transfer excess electrons to O_2_, ultimately reducing it to water in a process known as the Mehler-like reaction [17]. Thus, FDPs serve as an important photoprotective mechanism by preventing over-reduction of the PETC and safeguarding PSI from subsequent damage. In particular, the Flv1/Flv3 hetero-oligomer plays a crucial role under fluctuating light intensities [2].

Various engineering strategies targeting the PETC have been explored to enhance the supply of electrons to heterologous enzymes involved in biotransformation. Among these efforts, FDPs have often been targeted, as they could function as competing electron sinks and diverting electrons away from the desired heterologous pathways [3, 11]. However, deletion of the Flv1/Flv3 hetero-oligomer has shown improvement in biotransformation efficiency only in dense cultures [3, 11, 46]. It is important to note that, recent studies have shown that FDP hetero-oligomers utilise Fd as their electron donor during light-dependent O_2_ reduction (photoreduction) in vivo [34, 36, 44]. In contrast, BVMOs and YqjM rely on NAD(P)H. In particular YqjM, as a strong electron sink, can effectively outcompete FDPs for electrons by maintaining the NADP^+^ substrate pool of FNR highly oxidised [23], making FDP deletion futile from a competition perspective. Beyond FDPs, deletion of other electron transport components, such as terminal oxidase, aa3-type cytochrome *c* oxidase (COX) and bidirectional hydrogenase, has shown improvement in systems expressing cytochrome P450 enzymes, which depend on reduced Fd as their electron donor [6, 25, 47]. These findings demonstrate the credibility of electron transport chain engineering; however, further research on engineered production strains, specifically under actual production conditions, is crucial to fully understand the metabolic and energetic impacts of heterologous electron sinks and guide the development of effective improvement strategies.

The physiological state of cyanobacterial cells can also affect biotransformation efficiency; therefore, cells harvested at different growth stages will likely perform differently. Inorganic carbon (Ci) availability affects the global proteome profile, including the expression of PETC components and modulates cellular metabolism [33]. Under an ambient CO_2_ level, cyanobacteria invest energy in the carbon concentrating mechanism (CCM) to increase intracellular Ci availability, which in turn impacts PETC function and the bioenergetic state of the cells. Furthermore, both CO_2_ and HCO_3_^−^ have a role in signalling [28]. While heterologous enzymes may benefit from enhanced photosynthetic efficiency under elevated CO_2_, increased CBB cycle activity also raises the demand for bioenergetic cofactors, NADPH and ATP, thus intensifying competition for NADPH. The interplay between native and heterologous enzymatic pathways and the effect of Ci availability on their catalytic efficiencies, remain to be fully explored.

In *Synechocystis*, changes in the light spectrum during growth, were shown to alter the redox state of PETC, the PSI/PSII ratio and growth rates [53], potentially affecting the distribution of electron flux between different metabolic sinks. However, the impact of different wavelengths on the activity of heterologous enzymes remains unexplored. Furthermore, BVMOs contain a FAD binding domain, and ene-reductases such as YqjM utilise a flavin mononucleotide (FMN) as a cofactor. Since FAD is involved in blue light perception and signalling [16, 37], illumination with different light spectra during growth and biotransformation may influence the activity of heterologous enzymes.

In this study, we used two representative BVMOs - Parvi and Xeno and one ene-reductase YqjM, heterologously expressed in the wild-type cyanobacterium *Synechocystis* (Syn) and in the Flv1 deletion strain (ΔFlv1). We characterised their biotransformation performance under various CO_2_ conditions and light qualities. We focused on the availability of photosynthetically produced NADPH and O_2_ to identify possible bottlenecks using biophysical, biochemical and real-time gas exchange measurements. To quantify BVMO-specific O_2_ uptake, we calculated a corrected O_2_ uptake (CorrO_2_), which accounts for the substrate-induced effects on cellular physiology. Our data show that tailored strategies to improve the biotransformation activity for each enzyme are necessary. A “one-size-fits-all” approach is unlikely to be effective for different biotransformation applications.

## Materials and methods

### Strains and culturing conditions

The following *Synechocystis* sp. PCC 6803 strains were used in this study: Syn [51], ΔFlv1 [17], Syn with expressed Parvi (Syn::Parvi, Erdem et al., [11], Syn with expressed Xeno (Syn::Xeno, this work), ΔFlv with expressed Parvi or Xeno (ΔFlv1::Parvi or ΔFlv1::Xeno, respectively, Erdem et al., [11], Syn or ΔFlv1 with expressed YqjM (Syn::YqjM or ΔFlv1::YqjM, respectively [3]. Syn::Xeno was constructed using a plasmid described in Erdem et al. [11]. The plasmid was transformed into Syn, the gene was integrated into neutral site *slr0168*, and the strain segregation was verified by colony PCR using primers annealing in the homologous flanking regions (Fig. S1). All enzymes were expressed under the control of the P_cpcB_ promoter and tagged with an N-terminal His­tag.

Photoautotrophic cultivation was performed in 30 mL of BG‑11 medium (20 mM HEPES, pH 7.5) in 100 mL Erlenmeyer flasks under orbital shaking at 115 rpm. The cells were cultivated in AlgaeTron AG 130 (PSI, Czechia) at 30 °C under ambient CO_2_ (0.04%, LC) or elevated CO_2_ (3%, HC) conditions, with illumination at 150 µmol_photons_ m^‑2^ s^‑1^ (LED white light). When specified, cultivation was performed under LED white light enriched with red and blue wavelengths (W + R/B, Fig. S2). Precultures were grown in the same conditions as experimental cultures (LC or HC; 30 °C; 150 µmol_photons_ m^‑2^ s^‑1^; starting OD_750_ = 0.1). Kanamycin (50 µg mL^− 1^) and chloramphenicol (10 µg mL^− 1^) were used in precultures and stock cultures according to strains’ resistance cassettes. No antibiotic was present in the experimental cultures.

### Light-driven whole-cell biotransformation

Cells from the experimental cultures were harvested in the logarithmic growth phase (OD_750_ ~ 0.8 in LC or OD_750_ ~ 1.8 in HC) and resuspended in fresh BG-11 (pH 7.5) to the final cell density OD_750_ = 10. The biotransformation reaction was performed with LC cultures under LC conditions and with HC cultures under HC conditions. The biotransformation reaction was carried out in a reaction volume of 5 mL in a glass vial (V = 70 mL), sealed with a gas-tight rubber cap, at 30 °C under 300 µmol_photons_ m^‑2^ s^‑1^ of white illumination or W + R/B illumination when specified. The vials were laid horizontally on a shaker set to orbital shaking at 115 rpm. The reaction was initiated by adding 5 mM cyclohexanone (1 M stock in ethyl acetate) for strains expressing BVMO or 10 mM 2-methylmaleimide (2-MM, 100 mM stock in BG­11) for YqjM. At set intervals, 300 µL samples were taken, quickly frozen in liquid N_2_ and stored at − 20 °C before analysis by gas chromatography (GC). Specific activity was calculated using the ε-caprolactone concentration at 45 min timepoint (BVMOs) or 2-methylsuccinimide (2-MS) concentration at the 15 min timepoint (YqjM), and normalised to dry cell weight (DCW, OD_750_ = 10 ~ 2.4 g_DCW_ L^− 1^).

For the physiological experiments, the samples were adjusted to Chl *a* concentration of 10 µg mL^− 1^ (OD_750_ = 2–2.5), and cyclohexanone was added to a final concentration of 1.25 mM. The samples were taken after incubation for 50 min under 300 µmol_photons_ m^‑2^ s^‑1^ white illumination. For NADPH measurements the samples were further diluted 1:1 with BG-11 to reach Chl *a* concentration of 5 µg mL^− 1^ and dark-adapted for 10 min prior measurement.

### Gas chromatography

Samples were extracted using a three-step liquid-liquid extraction with ethyl acetate. The organic phase was dried over anhydrous MgSO_4_ and analysed using a GC-2010 Pro gas chromatograph (Shimadzu, Japan) equipped with an HP-5MS column (30 m × 0.25 mm, 5%-Phenyl-methylpolysiloxane column; 19091 S-133, Agilent, USA). Splitless injection mode was used. Compounds were separated at 35 °C (hold 3 min), 200 °C (hold 3 min, 10 °C min^− 1^) and 300 °C (hold 3 min, 25 °C min^− 1^). The linear velocity of the carrier gas (nitrogen) in the column was 11 cm s^− 1^. Acetophenone was used as the internal standard. Calibration was performed with known amounts of 2-MM and 2-MS for YqjM or cyclohexanone, ε­caprolactone and cyclohexanol for BVMOs.

### NAD(P)H fluorescence

NAD(P)H fluorescence was measured with the NADPH/9-AA module [42] for DUAL-PAM-100 (Walz, Germany). NAD(P)H fluorescence changes were monitored during a 10 s dark period followed by 180 s red actinic light (AL, 300 µmol m^− 2^ s^− 1^) and a subsequent 1 min dark period. The difference in NAD(P)H fluorescence between the initial dark period and shortly after the onset of illumination is shown in the figures as light-induced NADPH accumulation.

### Membrane Inlet mass spectrometry (MIMS)

The in vivo fluxes of ^16^O_2_ (m/z = 32), ^18^O_2_ (m/z = 36), and CO_2_ (m/z = 44) were followed by a built-in-house MIMS setup consisting of a modified oxygen electrode chamber (Hansatech Instruments Ltd., UK) sealed by a thin gas-permeable Teflon membrane (YSI Inc., USA) and connected to a MS (Prima PRO, Thermo Scientific, USA) via vacuum line. The final Chl *a* concentration was ~ 10 µg mL^− 1^, and the total dissolved Ci concentration was adjusted to 1.5 mM by adding NaHCO_3_. The illumination sequence was set to 2-5-3 min of dark-light-dark, respectively. After 50 min biotransformation, the samples were transferred into the MIMS sample chamber kept at 30 °C, briefly flushed with N_2_ to partially remove ^16^O_2_, enriched by ^18^O_2_ to reach equal isotopic abundance, and gas fluxes were measured based on mass signals. The gas exchange rates (gross and net O_2_ evolution, O_2_ uptake, CO_2_ consumption) were calculated as reported by Beckmann et al. [5].

The BVMO-specific corrected O_2_ uptake rate (CorrO_2_) was calculated by subtracting the O_2_ uptake rate of samples without cyclohexanone (O_2 Syn::Xeno_ – _S_) from that of samples with cyclohexanone (O_2 Syn::Xeno +S_). Furthermore, the O_2_ uptake rate of the background strain (Syn for Syn::Xeno and Syn::Parvi, ΔFlv1 for ΔFlv1::Xeno and ΔFlv1::Parvi) was subtracted. Equation 1 depicts the calculation example for Syn::Xeno.

1 $$\begin{gathered} CorrO_{{2_{{Syn::{\kern 1pt} Xeno}} }} = \left( {O_{{2_{{Syn::{\kern 1pt} Xeno + S}} }} - O_{{2_{{Syn::{\kern 1pt} Xeno - S}} }} } \right) \hfill \\ - \left( {O_{{2_{{Syn + S}} }} - O_{{2_{{Syn - S}} }} } \right) \hfill \\ \end{gathered} $$

### Protein extraction and Immunoblotting

Total protein extracts were isolated as previously described by Zhang et al. [54], separated by sodium dodecyl sulphate-polyacrylamide gel electrophoresis (SDS-PAGE) using Bio-Rad Mini-PROTEAN TGX 4–15% precast gels and transferred onto polyvinylidene fluoride membranes. The membranes were probed with a commercial Anti-polyHistidine − Peroxidase antibody (Sigma-Aldrich, USA), and detection was performed using Amersham ECL (GE Healthcare, USA).

### Statistical analysis

Analysis and visualisation were conducted using R statistical software [10]. Two-way ANOVA or unpaired t-tests were used to assess statistical significance. Tukey’s test was used to compare the means of different groups with each other, with *p* < 0.05 as the significance cut-off.

## Results

### O_2_ or NADPH are not limiting for syn::xeno’s activity under specific experimental conditions

**Fig. 1:**
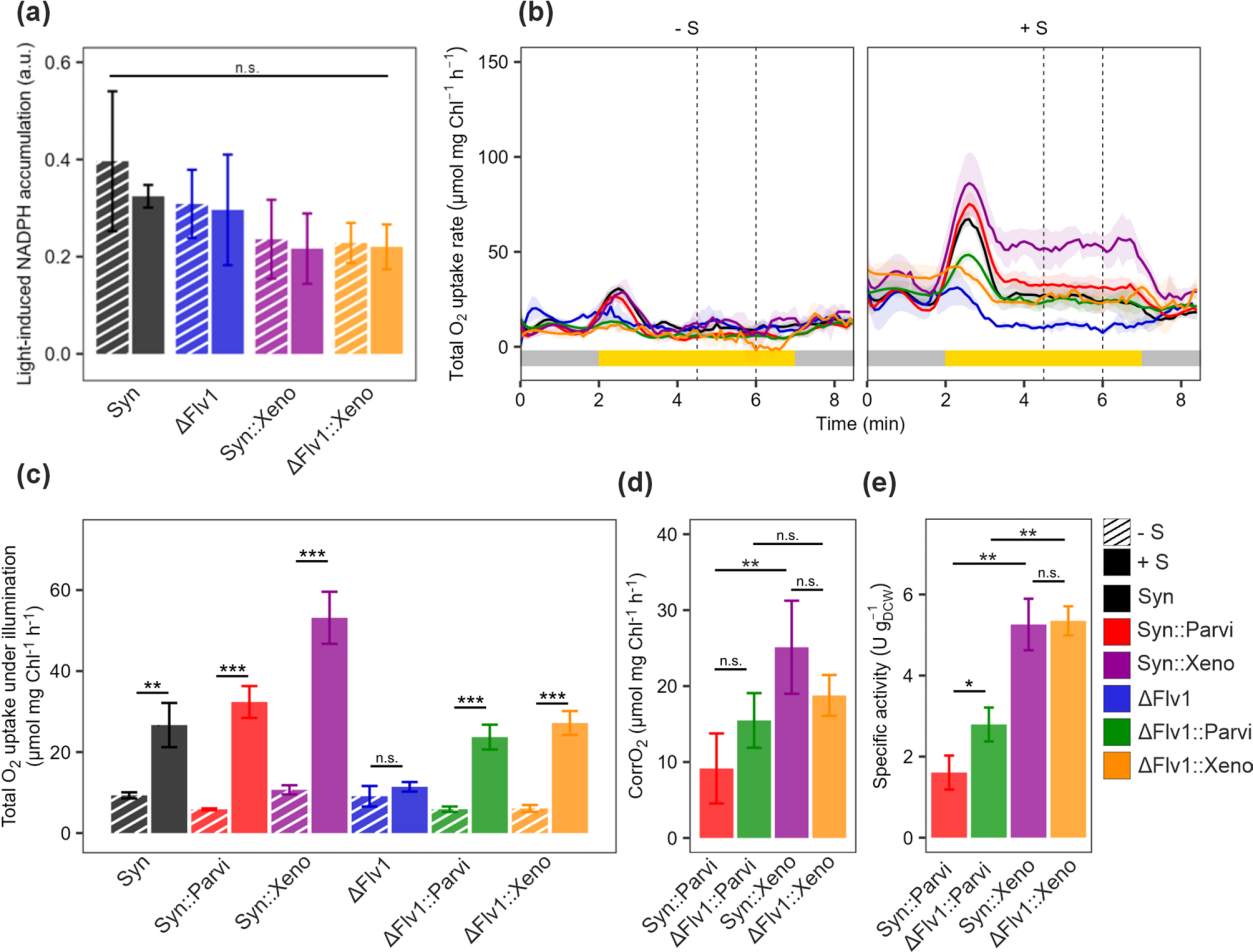
Characterisation of BVMO-expressing strains under ambient air. The samples for physiological measurements were taken after 50 min of biotransformation (OD_750_ = 2-2.5). **a** The light-induced NADPH level determined as the difference between NAD(P)H fluorescence before and shortly after the onset of illumination. **b** The O_2_ uptake kinetics. The vertical dashed line represents steady-state (4.5–6 min). **c** The total steady-state O_2_ uptake rate under illumination. **d** CorrO_2_ of BVMO-expressing strains. (e) Specific activity calculated from the 45-min timepoint of the biotransformation reaction (OD_750_ = 10) based on the concentration of ε­-caprolactone (Fig. S4). The column bars represent Mean ± Standard Deviation (SD). Black - Syn, blue - ΔFlv1, red - Syn::Parvi, green - ΔFlv1::Parvi, magenta - Syn::Xeno, orange - ΔFlv1::Xeno, striped - - S, full - + S. Statistical significance was tested by one-way ANOVA or t-test, *≤0.05, **≤0.01, ***≤0.001. P values can be found in Table S1

Characterising the strain used in biotransformation under defined production conditions is necessary to ensure consistent, reproducible performance and to enable targeted optimisation of productivity. We selected white light illumination and ambient air (0.04% CO_2_, LC) as the standard biotransformation conditions since they are commonly used in laboratory and biotechnological applications. Since BVMO activity requires O_2_ as a substrate and NADPH as a cofactor, we assessed the availability of both during biotransformation under standard conditions. To test the level of photosynthetically produced NADPH, we used two strains: Syn::Xeno, which expresses the high-turnover recombinant enzyme Xeno [11] in the wild-type background and ΔFlv1::Xeno strain, expressing Xeno in the Flv1 deletion background. Since Flv1/3 hetero-oligomers channel excess electrons from photosynthetically reduced Fd to O_2_, eliminating these proteins has been used as a strategy to redirect more electrons toward bio-production with various results. Light-induced NADPH accumulation in Syn::Xeno and ΔFlv1::Xeno strains remained similar in the presence (+ S) and absence (− S) of the substrate cyclohexanone (Fig. 1a), indicating that NADPH supply was not limiting under the studied conditions (after 50 min of biotransformation at OD_750_ = 2–2.5). Cyclohexanone also had no significant effect on light-induced NADPH accumulation in the background strains Syn and ΔFlv1 (Fig. 1a).

To investigate oxygen flux dynamics and assess BVMO-mediated O_2_ uptake in strains expressing BVMO, we monitored real-time gas exchange using MIMS and the ^18^O_2_ isotope, allowing us to differentiate gross O_2_ evolution by PSII from O_2_ uptake by the cellular metabolism and BVMO under illumination. These experiments were conducted after 50 min of active biotransformation (OD_750_ = 2–2.5). We first tested BVMO-expressing Syn::Xeno, Syn::Parvi, ΔFlv1::Xeno, ΔFlv1::Parvi and their respective background strains (Syn and ΔFlv1) under experimental conditions mimicking the biotransformation set-up, but in the absence of substrate (-S). Upon transitions from dark to light or from low to high light intensity, *Synechocystis* cells typically exhibit strong O₂ uptake during the initial minutes, which then transitions into a quasi-stable phase of O_2_ uptake [40]. This light-induced O_2_ uptake is primarily attributed to the activity of FDPs, however minor contributions from other O_2_-consuming components (e.g., respiratory terminal oxidases) cannot be fully excluded under specific conditions [12]. Interestingly, under these experimental conditions, only transient O_2_ uptake was observed upon illumination in all strains. This is most likely due to the fact that the cultures were grown under a high light intensity, and the gas-exchange experiments were conducted under the same light intensity as during biotransformation. As a result, overall O_2_ uptake during illumination remained low (Fig. 1b). A similar trend was observed when the cells were grown at 50 µmol_photons_ m^‑2^ s^‑1^, and MIMS measurements were done at the same light intensity [2, 38]. The ΔFlv1 strain served as an appropriate control for assessing the BVMO-specific O_2_ uptake under illumination, as this mutant lacks the light-induced O_2_ uptake upon dark-to-light transition, thereby preventing signal masking by the Mehler-like reaction. All BVMO-expressing strains exhibited a significant increase in total O_2_ uptake in the presence of cyclohexanone, indicating active biotransformation (Fig. 1c, +S). Surprisingly, cyclohexanone addition significantly increased the total O_2_ uptake rate under illumination also in the background Syn but had no effect in ΔFlv1 (Fig. 1c). With the concentration and in the conditions used here, cyclohexanone had no detectable effect on gross O_2_ evolution or CO_2_ fixation in any of the strains (Fig. S4).

To differentiate BVMO-specific O_2_ uptake and account for the substrate-induced effects in the background strains, we calculated the “BVMO-specific corrected O₂ uptake” (CorrO_2_) in the BVMO-expressing strains. This was necessary due to the different responses of Syn and ΔFlv1 strains to cyclohexanone, where Syn showed increased total O_2_ uptake, while ΔFlv1 did not (Fig. 1c). First, we determined the substrate-specific O_2_ uptake under biotransformation conditions as the difference between the total O_2_ uptake with substrate (+ S) and without substrate (− S) for each strain. We then subtracted the substrate-specific O_2_ uptake of the respective background strain (Syn and ΔFlv1) from the substrate-specific O_2_ uptake of BVMO-expressing strains (Eq. 1, see Methods). The calculated CorrO_2_ value thus corresponds to the O_2_ uptake attributed to the BVMO activity. Although net O_2_ evolution decreased significantly in Syn::Xeno, ΔFlv1::Parvi, and ΔFlv1::Xeno, it remained high in all BVMO-expressing strains (Fig. S3, +S); therefore, O_2_ availability is likely not a limiting factor under these specific experimental conditions.

Next, we followed the conversion of cyclohexanone into ε-caprolactone over five hours to assess the performance of BVMO-expressing strains under 0.04% CO_2_, white light, and OD_750_ = 10 conditions (Fig. S4) and calculated specific activity at the 45 min time point (see Methods). In line with previously published results [11], Syn::Xeno showed 3-fold higher specific activity than Syn::Parvi (Fig. 1e), consistent with the elevated CorrO2 observed in Syn::Xeno (Fig. 1d). We also tested strains expressing BVMO in the Flv1 deletion background (ΔFlv1::Parvi and ΔFlv1::Xeno). Although a significant increase in specific activity was observed in ΔFlv::Parvi (2.78 ± 0.42 U g_DCW_^−1^) compared to Syn::Parvi (1.61 ± 0.42 U g_DCW_^−1^), no difference was observed between ΔFlv::Xeno and Syn::Xeno (Fig. 1e), contrary to previous publications [11]. After five hours, Syn::Xeno reached a slightly higher ε-caprolactone concentration than ΔFlv1::Xeno, and ΔFlv1::Parvi was marginally higher than Syn::Parvi (Fig. 1d, Fig. S4).

**Fig. 2:**
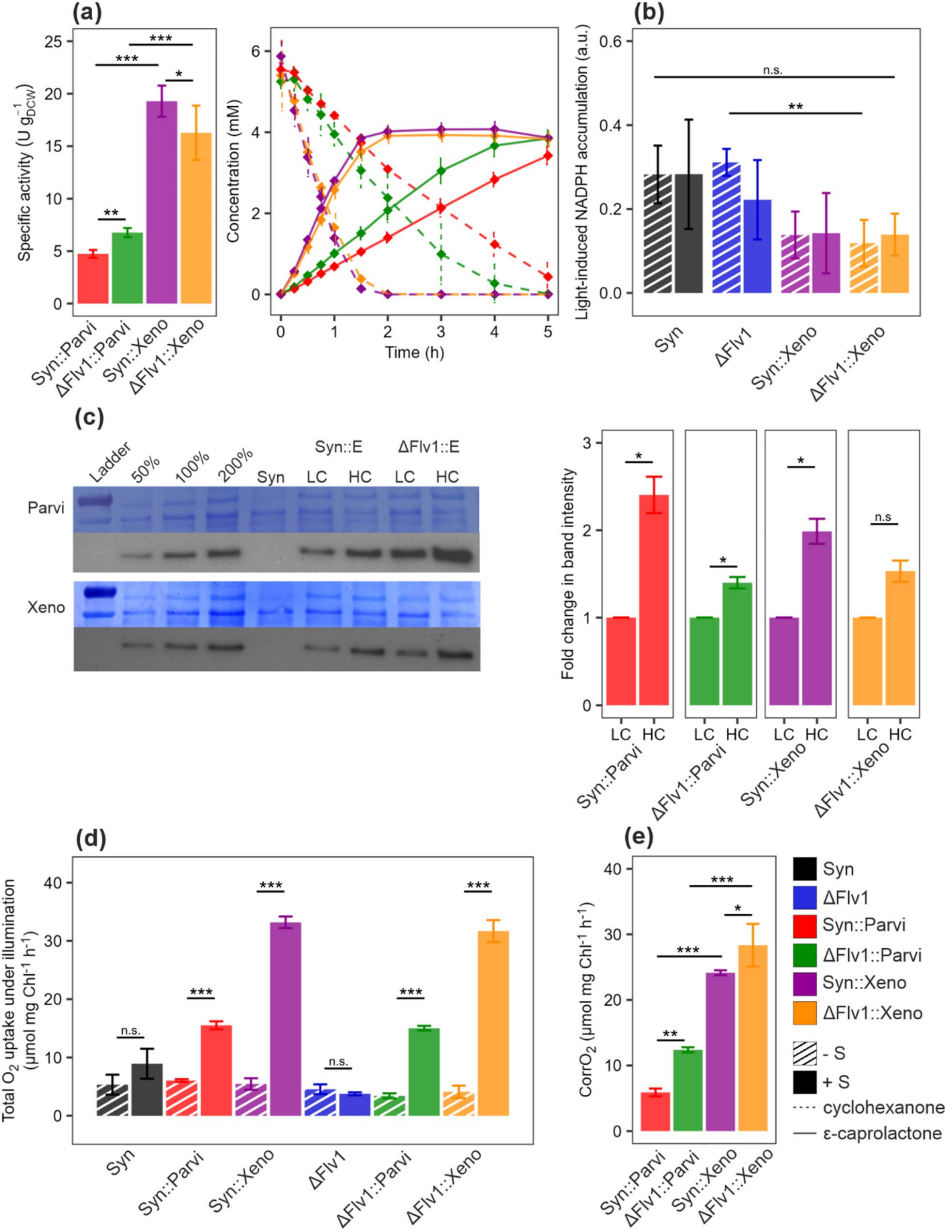
Characterisation of strains expressing BVMO under HC conditions. **a** Specific activity calculated from the 45-min timepoint of the biotransformation (at OD_750_ = 2-2.5) based on the concentration of ε-caprolactone and the cyclohexanone and ε-caprolactone concentration during biotransformation over 5 h. **b** The light-induced NADPH level determined as the difference between NAD(P)H fluorescence measured before and after the onset of illumination. **c** Immunodetection of BVMO enzymes with an anti-His antibody and relative protein abundance changes compared to the LC sample. A representative replicate is shown; the remaining replicates are displayed in Fig. S7. Syn::E - *Synechocystis* expressing Parvi or Xeno, ΔFlv1::E - ΔFlv1 strain expressing Parvi or Xeno. **d** The total O_2_ uptake under illumination with and without substrate in the media. **e** CorrO_2_ of BVMO-expressing strains. The column bars represent Mean ± SD (or Standard Error of the Mean - SEM in c). Black - Syn, blue - ΔFlv1, red - Syn::Parvi, green - ΔFlv1::Parvi, magenta - Syn::Xeno, orange - ΔFlv1::Xeno, striped - -S, full - +S. Statistical significance was tested by one-way ANOVA or t-test, *≤0.05, **≤0.01, ***≤0.001. P values can be found in Table S1

The protein abundance of heterologous enzymes has been suggested as a potential limiting factor for biotransformation activity [24]. To assess enzyme levels, we performed immunodetection of the N-terminal His-tag (Fig. 2c; Fig. S5). ΔFlv1::Xeno showed approximately 50% higher BVMO accumulation compared to Syn::Xeno, which did not correlate with the increased measured activity, while ΔFlv1::Parvi showed a 200% increase in protein accumulation compared to Syn::Parvi consistent with the observed activity (Fig. 1e, Fig. S4).

### Growth and biotransformation under elevated CO_2_ enhance bvmos’ conversion rate

*Synechocystis* undergoes profound metabolic changes when grown under elevated CO_2_ levels, showing improved growth and photosynthetic activity [33, 40]. Under HC (3%) conditions, all four BVMO-expressing strains exhibited significantly higher specific activity. In particular, Syn::Xeno showed a 4-fold increase, reaching 19.61 ± 0.35 U g_DCW_^−1^ compared to 5.26 ± 0.64 U g_DCW_^−1^ in LC. Similarly, Syn::Parvi exhibited a 3-fold increase, from 1.61 ± 0.42 in LC to 4.74 ± 0.38 U g_DCW_^−1^ in HC (Fig. 2a). Syn::Xeno also achieved a complete conversion of 5 mM cyclohexanone within less than two hours (Fig. 2a) compared to more than five hours in LC (Fig. 1e). As in LC, ΔFlv1::Parvi had higher specific activity than Syn::Parvi (6.77 ± 0.43 U g_DCW_^−1^ vs. 4.74 ± 0.38 U g_DCW_^−1^, respectively). Interestingly, ΔFlv1::Xeno exhibited lower specific activity than Syn::Xeno (17.02 ± 1.33 U g_DCW_^−1^ vs. 19.61 ± 0.35 U g_DCW_^−1^, respectively).

The *cpcB* gene, whose truncated promoter is used in our study, is upregulated during growth in elevated CO_2_ concentration [33]. In line with this, as well as the increase in specific activity, cells grown in HC conditions showed strong BVMO expression, with a 50–150% increase in protein abundance relative to LC samples (Fig. 2c). The increased abundance of BVMO protein observed in ΔFlv1 backgrounds compared to Syn under LC conditions was also evident under HC, although the difference was no longer statistically significant (Fig. 2c, Fig. S5). Notably, ΔFlv1 exhibits significant transcriptomic reprogramming relative to Syn (64 genes up- or down-regulated; Mustila et al., [32].These altered expression patterns and lack of Mehler-like reaction in ΔFlv1 could influence the expression and stability of heterologous enzymes in both conditions.

As in LC conditions, no difference was observed in NAD(P)H fluorescence changes upon illumination between (+ S) and (− S) samples of each strain grown in HC (Fig. 2b) suggesting sufficient NADPH production by photosynthetic light reactions to support biotransformation. However, strains expressing Xeno demonstrated lower light-induced NADPH accumulation compared to their respective background strain, with the difference being significant for ΔFlv1::Xeno (Fig. 2b, -S). This may be due to the higher abundance of recombinant protein. In contrast to LC, the effect of cyclohexanone on the total O_2_ uptake rate under illumination in the background strains Syn and ΔFlv1 was negligible under HC conditions (Fig. 2d). The difference in total O_2_ uptake under illumination between Xeno and Parvi was more pronounced under HC, with Xeno showing a 2-fold higher steady-state total O_2_ uptake rate and a 5-fold higher CorrO_2_. Furthermore, the CorrO_2_ in ΔFlv1::Parvi is 2-fold higher than in Syn::Parvi under HC. ΔFlv1::Xeno also showed higher CorrO_2_ than Syn::Xeno despite slightly lower specific activity (Fig. 2e, a). However, it should be noted that these measurements were performed at different cell densities. The photosynthetic gross O_2_ evolution was slightly higher in HC compared to LC (for example in Syn: 210.18 ± 14.68 µmol mg_Chl_^−1^ h^− 1^ and 177.68 ± 11.85 µmol mg_Chl_^−1^ h^− 1^ respectively; *P* = 0.04; Fig. S6).

### Light spectrum enriched in blue and red wavelengths enhances biotransformation under ambient CO_2_ level

**Fig. 3:**
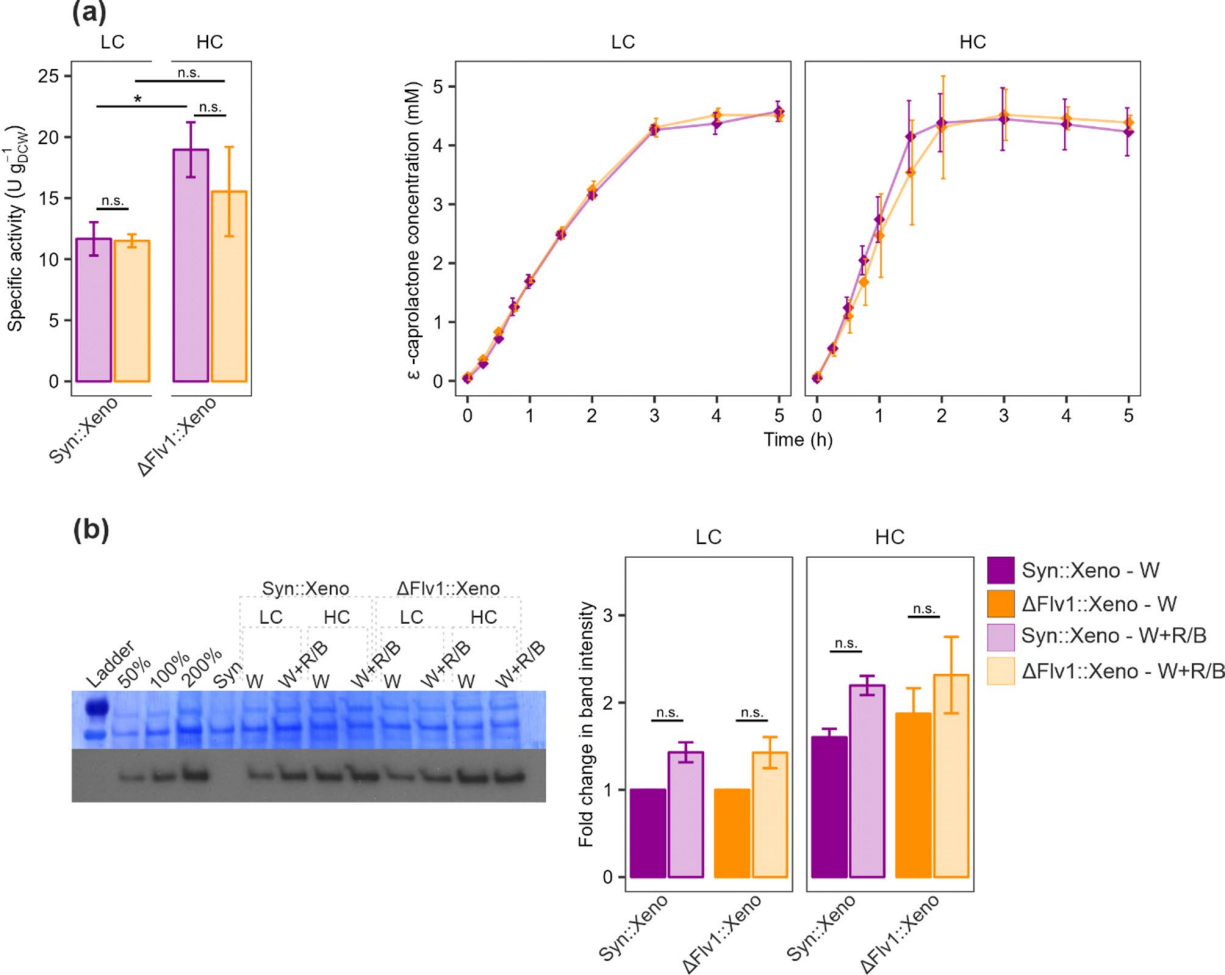
Effects of W + R/B illumination in LC or HC conditions on Xeno-expressing strains. **a** Specific activity calculated from the 45-min timepoint of the biotransformation based on the concentration of ε-caprolactone. And the ε­-caprolactone concentration during biotransformation over 5 h. **b** Immunodetection of BVMO enzymes with anti-His antibody and relative protein abundance change in relation to the LC sample. A representative replicate is shown with the remaining replicates in Fig. S7. The column bars represent Mean ± SD (**a**) or SEM (**b**). W - white light, W + R/B - white light enriched with red and blue wavelengths, magenta - Syn::Xeno, orange - ΔFlv1::Xeno, full colour - W, light colour - W + R/B. Statistical significance was tested by t-test, *≤0.05, P values can be found in Table S1

In addition to elevated CO_2_ levels, we compared the biotransformation performance of Syn::Xeno and ΔFlv1::Xeno cultivated under broad-spectrum white light and white light enriched in blue and red wavelengths (W + R/B) with biotransformation conducted under the same conditions (Fig. S2). Cells grown under W + R/B and LC conditions demonstrated significantly higher biotransformation performance, with Syn::Xeno reaching a specific activity of 11.66 ± 1.37 U g_DCW_^−1^, a 2-fold increase compared to white light and LC (Figs. 1e and 3a). Interestingly, using W + R/B under HC conditions did not further increase specific activity, which reached 18.97 ± 2.25 U g_DCW_^−1^, similar to white light in HC (19.61 ± 0.35 U g_DCW_^−1^; Figs. 2a and 3a).

The protein abundance of Xeno showed only a slight, but statistically non-significant increase under W + R/B illumination compared to white light in both LC and HC conditions (Fig. 3b, Fig. S7). However, since the specific activity of Syn::Xeno and ΔFlv1::Xeno increased only under W + R/B combined with LC conditions, the unchanged biotransformation efficiency under W + R/B with HC suggests that protein accumulation is not the limiting factor, instead another constraint has likely been reached.

### YqjM does not respond to increase in CO_2_ levels

**Fig. 4:**
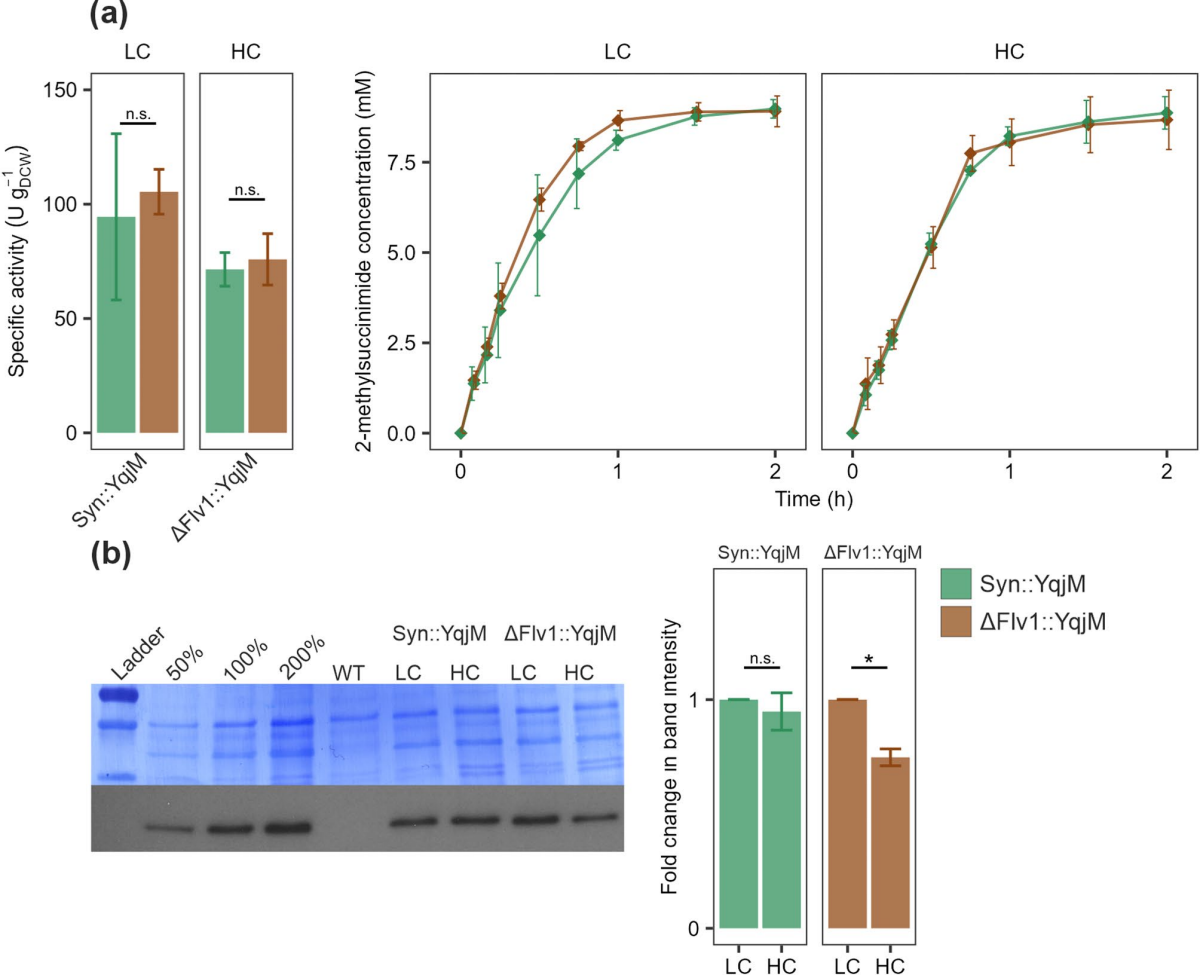
Characterisation of Syn::YqjM and ΔFlv1::YqjM in LC and HC conditions. **a** Specific activity calculated from the 15-minute timepoint of the biotransformation based on the concentration of 2-MS. And the 2-MS concentration during biotransformation over 2 h. **b** Immunodetection of YqjM enzyme with an anti-His antibody and measurement of relative protein abundance change in relation to the LC sample. A representative replicate is shown with the remaining replicates in Fig. S7. The column bars represent Mean ± SD (or SEM in **b**). Seagreen - Syn::YqjM, brown - ΔFlv1::YqjM. Statistical significance was tested by one-way ANOVA, * ≤ 0.05. P values can be found in Table S1

To assess whether the increase in specific activity and enzyme abundance observed with BVMOs in HC conditions is transferable to different enzyme types, we used a strain expressing the NAD(P)H-dependent ene-reductase YqjM grown under white light and LC and HC conditions. Contrary to BVMOs, the elevated CO_2_ concentration during growth and biotransformation did not increase YqjM activity. Instead, a slight decrease was observed in HC conditions (Fig. 4a; Fig. S7). This reduction also aligned with lower YqjM enzyme accumulation, which was statistically significant in ΔFlv1::YqjM under HC compared to LC conditions, as detected by immunodetection with anti-His antibody (Fig. 4b). This was surprising since all three enzymes share the same P_cpcB_ promoter and their integration locus is the same. It is possible that the expression of YqjM is further affected by other mechanisms involved in protein and RNA stability and degradation. However, the exact cause behind these results is unknown.

## Discussion

Cyanobacteria are a promising platform for the solar-driven production of fine and commodity chemicals. However, despite recent advances, current production yields remain insufficient to compete with conventional production processes. Achieving market viability will require further improvements in metabolic engineering and photobioreactor design, process engineering and upscaling [9]. Our study highlights the importance of first optimising cultivation and biotransformation conditions before implementing additional strain engineering for improved efficiency. Notably, we demonstrate that BVMO activity significantly increases under elevated CO_2_ concentrations in all studied strains, with Syn::Xeno showing a 4-fold increase in specific activity (Fig. 2a). Additionally, under air-level CO_2_, the specific activity was doubled when cells were cultivated under W + R/B compared to a broad-spectrum white light (Figs. 1e and 3a).

Assessing the limiting factor of the selected enzymatic reaction is crucial, as the availability of NADPH and O_2_ for BVMOs and NAD(P)H for YqjM can limit their activity. Our results showed that the light-induced NADPH accumulation remained unchanged in the presence or absence cyclohexanone in both LC and HC conditions (Figs. 1a and 2b), indicating that the NADPH turnover is sufficient to meet demand of BVMO (OD_750_ = 2-2-5), as suggested earlier [8]. Under HC conditions, Syn::Xeno achieved a specific activity of 19.61 ± 0.35 U g_DCW_^−1^, while the estimated light-induced NADPH production capacity is 106–212 U g_DCW_^−1^ [27, 49]. It is important to note that the expression of Xeno caused a decrease in light-induced NADPH accumulation already in the absence of cyclohexanone, especially in HC conditions (Figs. 1a and 2b). This suggests that expression of the heterologous BVMO enzyme can act as a significant NADPH sink even in the absence of its substrate. This could be due to the enzyme’s uncoupling effect, in which NADPH is consumed without substrate oxidation, leading to the release of H_2_O_2_ [18]. However, H_2_O_2_ production by uncoupled BVMO activity would involve consumption of O_2_, which may have been masked by rapid catalase-induced H_2_O_2_ scavenging and O_2_ release, since we did not detect it by MIMS (Figs. 1c, 2d). Alternatively, such high-level expression might disturb the spatial organisation of protein complexes associated with the thylakoid membrane, potentially slowing down electron transfer to NADP^+^, although unchanged gross O₂ production does not fully support this hypothesis. Lastly, BVMOs could use an endogeneous substrate, thus continuously consuming NADPH, even in the absence of added cyclohexanone. It is also important to note that while a strong electron sink that continuously drains NADPH may enhance photosynthetic efficiency, it can ultimately disrupt the ATP/NADPH balance and lead to cellular exhaustion during long-term processes. Therefore, including recovery phases without substrate addition is essential in prolonged production systems.

The availability of O_2_ is a major bottleneck for BVMO applications in heterotrophic hosts [9, 13]. In this context, cyanobacteria provide an interesting alternative due to their ability to generate O_2_ via photosynthesis. In our experiments, gross O_2_ evolution by Syn reached ~ 180 µmol mg_Chl_^−1^ h^− 1^ under LC, corresponding to ~ 50 U g_DCW_^−1^ and ~ 210 µmol mg_Chl_^­1^ h^− 1^under HC, corresponding to ~ 60 U g_DCW_^−1^. However, ΔFlv1 exhibited lower rates under both conditions (~ 140 µmol mg_Chl_^−1^ h^− 1^ and ~ 155 µmol mg_Chl_^­1^ h^­1^, respectively; Fig. S6). These values (obtained at OD_750_ = 2–2.5) are comparable to those reported by Zavřel et al. [52] at low densities (OD_680_ = 1). In our study, the total O_2_ uptake increased in strains expressing BVMO in the presence of cyclohexanone, compared to the control background strains (Figs. 1c and 2d). However, the net O_2_ evolution, calculated as the difference between gross O_2_ evolution and the total O_2_ uptake, remained high (Fig. S3; Fig. S8), indicating that under sufficient light availability (low cell densities), the photosynthetic O_2_ evolution could support higher BVMO activity. However, previously reported BVMO specific activities as high as 63 U g_DCW_^−1^ (Tüllinghof et al., 2023, biomass concentration 0.7–1.0 g_DCW_ L^− 1^, or OD_750_ = 3–4) suggest that BVMO activity can approach the upper limits of photosynthetic O_2_ supply. In dense cultures or large-scale photobioreactors, reduced light penetration due to self-shading, limits photosynthetic efficiency, consequently also O_2_ evolution and NADPH production. Given the stoichiometry of 2 NADPH per O₂ molecule produced in light reactions and the increased respiration under dark/dim light, O₂ availability may become the main limitation in such conditions. Therefore, engineering strains with enhanced O₂ production and improved extracellular O₂ diffusion into photobioreactor is essential to enable efficient oxyfunctionalisation at high cell densities and in scaled-up systems.

Substrate and product toxicity is a common challenge in biotechnological applications, particularly in biotransformation processes, where it can compromise cell viability and limit overall productivity. The substrate of YqjM (2-MM), has been shown to severely impair photosynthetic apparatus by affecting thylakoid membrane conductivity, preventing the build-up of proton motive force [23]. Toxic effects can sometimes be mitigated by improved photobioreactor design or substrate-feeding strategies, such as two-liquid phase systems [22] or by immobilisation of cells in biocompatible scaffolds [48]. However, in some cases, toxicity or side-effects can unintentionally yield beneficial effects. For instance, 2-MM, the substrate of YqjM [4], has been shown to inhibit the CO_2_ fixation via the CBB cycle, thus removing a major competing sink for NADPH [23]. In contrast, cyclohexanone does not affect CO_2_ fixation and O_2_ evolution (Fig. S3; Fig. S8), at least at the concentrations used in our study. In *Chlamydomonas reinhardtii*, 5mM cyclohexanone caused only a minor reduction in growth and gross O_2_ evolution [45].

Low heterologous gene expression can limit biotransformation activity; therefore, strong constitutive or inducible promoters are often used. Under a CO_2_-rich environment, BVMO expression under the P_cpcB_ promoter increased (Fig. 2c), followed by higher specific activity (Fig. 2a). This indicates that the protein level is the limiting factor for BVMOs in ambient air conditions, and strategies targeting modulation of expression could yield further improvements of biotransformation productivity. Furthermore, the accumulation of Parvi was significantly higher in ΔFlv1::Parvi than in Syn::Parvi (Fig. S5), likely explaining the observed increase in specific activity (Fig. 1e). A smaller increase in Xeno protein accumulation was also observed between ΔFlv1::Xeno and Syn::Xeno strains (Fig. S5), though without a corresponding change in specific activity (Fig. S4), suggesting that biotransformation performance depends not only on protein abundance but also, most likely, on other factors like enzyme localisation. Interestingly, YqjM abundance did not respond to high CO_2_ conditions (Fig. 4) despite using the same P_cpcB_ promoter, and its specific activity remained comparable. The observed differences in expression patterns may be due to limitations in the availability of substrates (e.g., ATP) required for protein synthesis. The lack of improvement in YqjM specific activity may be related not only to limited protein abundance, but also to the side effects of 2-MM, its substrate, on photosynthesis. Given that 2-MM inhibits CO₂ fixation [23], a substantial CO₂-driven enhancement in the availability of photosynthetic reducing equivalents cannot be expected. In *Synechococcus elongatus* PCC 7942, alcohol dehydrogenase (ADH) mediated whole-cell biotransformation improved under 0.5% CO_2_, likely due to enhanced NADPH regeneration [43]. Although ADH protein abundance was not quantified, changes in expression cannot be excluded as a contributing factor.

The light spectrum influences cyanobacteria’s physiology and photosynthetic performance [53]. Blue light, in particular, alters the transcription of photosynthesis-related genes [29] and, as it preferably excites PSI, can impair photosynthesis due to over-reduction of PETC [7, 31]. However, these effects were observed under narrow or single-wavelength illumination, while our study used broad-spectrum white light enriched with red and blue wavelengths, limiting direct comparison. We observed a significant improvement in biotransformation efficiency with both Syn::Xeno and ΔFlv1::Xeno under W + R/B illumination, but only in LC conditions (Fig. 3a). Under HC, cells grown in W + R/B performed comparably to those grown in broad white light (Figs. 2a and 3a). The protein abundance of Xeno was not significantly altered, suggesting that the observed improvement under W + R/B is not related to increased protein levels. Instead, it suggests the involvement another mechanism that may become limiting under HC conditions. Notably, violet light was reported to enhance isoprene production in *Synechocystis* [39] underlining light quality as an important factor in cyanobacterial biotechnological applications. Furthermore, the use of broad-spectrum light with specific wavelength enrichments could offset the adverse effects of monochromatic illumination [7, 29, 53]. These findings highlight the need for systematic characterisation of the effects of light spectra on cyanobacterial physiology and in biotransformation setups. In this regard, determining the action spectrum of each recombinant biotransformation enzyme is essential for optimising biotransformation processes. However, the use of lights with unique spectra could increase the upfront production costs due to their higher cost.

Targeted deletion of alternative electron pathways is a common strategy to improve biotransformation yields [3, 11, 25, 46]. This has been motivated by the idea of removal of competing electron sinks, increasing the availability of reducing power for the heterologous enzyme [6, 47]. However, recent findings suggest that a strong heterologous sink can outcompete native electron sinks and alternative electron pathways [23]. Indeed, deleting FDPs in *Synechococcus* sp. PCC 7002 did not improve P450 activity [41]. In our study, the deletion of Flv1 proved beneficial only in the case of Parvi, but instead of electron redistribution, this was likely caused by a 3-fold increase in the protein accumulation (Figs. 1a and 2a; Fig. S4; Fig. S5). This effect may also reflect differences in wild-type backgrounds. ΔFlv1::Parvi and Syn::Parvi derive from different strains, unlike Syn::Xeno and ΔFlv1::Xeno, which share the same background. These findings highlight that the host strains can significantly affect heterologous expression, making strain selection a key consideration for biotechnological applications.

## Conclusions

This study underscores the importance of characterising both the heterologous enzymes and host strains for light-driven whole-cell biotransformation applications under production conditions. We observed significant improvements in BVMO activity under 3% CO_2_ conditions, while the same conditions had no effect on the NAD(P)H-dependent ene-reductase YqjM. Similarly, light spectra enriched in red and blue wavelengths benefited BVMO activity under ambient air, but no additional benefit was observed when combined with 3% CO_2_. These findings highlight the complex interplay between light, carbon availability and enzyme-specific responses. They underscore the need to individually optimise light-driven whole-cell biotransformations, as production strains exhibit distinct physiological responses depending on the environmental conditions and the enzyme expressed. A deeper understanding of the interactions between heterologous enzymes and cyanobacterial metabolism is essential to fully exploit their biotechnological potential. Therefore, future efforts should focus on unrevealing the regulatory networks of photosynthesis, the interplay between heterologous and native pathways, and the development of optimised and novel enzymes to advance cyanobacteria as an efficient and robust chassis for solar fuels and chemicals.

## Supplementary Information

Additional file 1.

## Data Availability

The datasets analysed during the current study are available from the corresponding author upon reasonable request.
