# Supplementary material for: Optimising CO2 level and light quality for enhanced whole-cell biotransformation reactions in Synechocystis sp. PCC 6803
Source: Microb Cell Fact. 2025 Aug 31;24:198. doi: 10.1186/s12934-025-02828-4 (PMC12398983; doi:10.1186/s12934-025-02828-4)

*Supporting Information*

**Optimising CO_2_ level and light quality for enhanced whole-cell biotransformation reactions in *Synechocystis* sp. PCC 6803**

Authors list: Michal Hubáček,^1^ Lauri Nikkanen,^1^ Yagut Allahverdiyeva^1, *^

Affiliation: ^1^*Molecular Plant Biology unit, Department of Life Technologies, University of Turku, Finland*

*Authors email*

Michal Hubáček - [mihuba@utu.fi](mailto:mihuba@utu.fi)

Lauri Nikkanen - [lenikk@utu.fi](mailto:lenikk@utu.fi)

* Corresponding: Yagut Allahverdiyeva - [allahve@utu.fi](mailto:allahve@utu.fi)

*Content*

**Figure S1.** Segregation check for Syn::Xeno.

**Figure S2.** Light spectra comparison of White and W+R/B illumination and the total energy calculation.

**Figure S3.** Gas exchange kinetics in the absence (- S) and presence (+ S) of C-one in Syn::Xeno and ΔFlv1::Xeno in LC conditions (0.04 % CO_2_).

**Figure S4.** The concentration of cyclohexanone and ε-caprolactone over the course of 5 h in LC.

**Figure S5.** Relative protein abundance changes of Parvi, Xeno or YqjM in LC vs HC conditions.

**Figure S6.** Gross O_2_ evolution in LC compared to HC conditions in the absence (- S) and presence (+ S) of cyclohexanone in Syn::Xeno and ΔFlv1::Xeno.

**Figure S7.** Immunodetection of BVMO enzymes and YqjM with an anti-His antibody.

**Figure S8.** Gas exchange kinetics in the absence (- S) and presence (+ S) of C-one in Syn::Xeno and ΔFlv1::Xeno in HC conditions (3 % CO_2_).

**Table S1.** Results of statistical tests.

**Supplementary Figures**


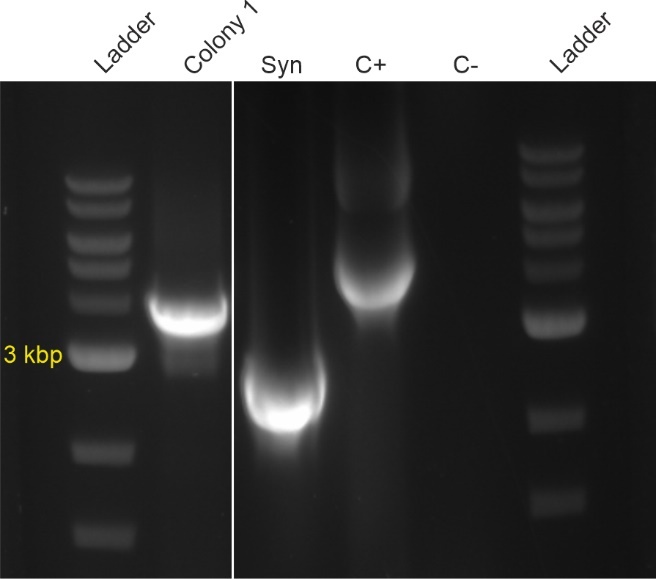


Figure S1. Segregation check for Syn::Xeno. The gel shows Colony 1 which was used in this study. The photo was modified to exclude 9 other colonies tested in the same experiment. Primers – FW: TGGCCCTGGACAGTCAGGAATG, REV: GCTGCATGTTGGGACTGGAGAC. Syn – WT genomic DNA, C+ - positive control (SynRekB_P_cpcB_BVMO_Xeno_ plasmid), C- - negative control (MQ water).


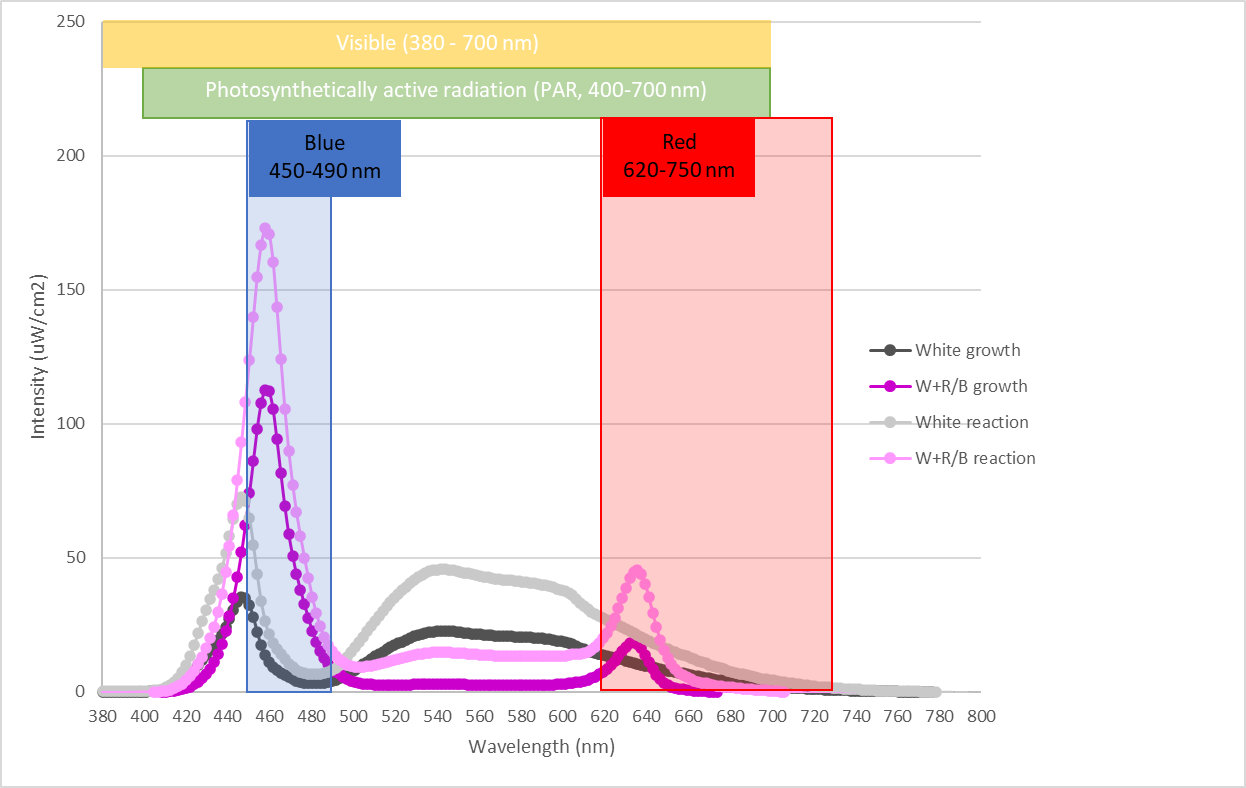

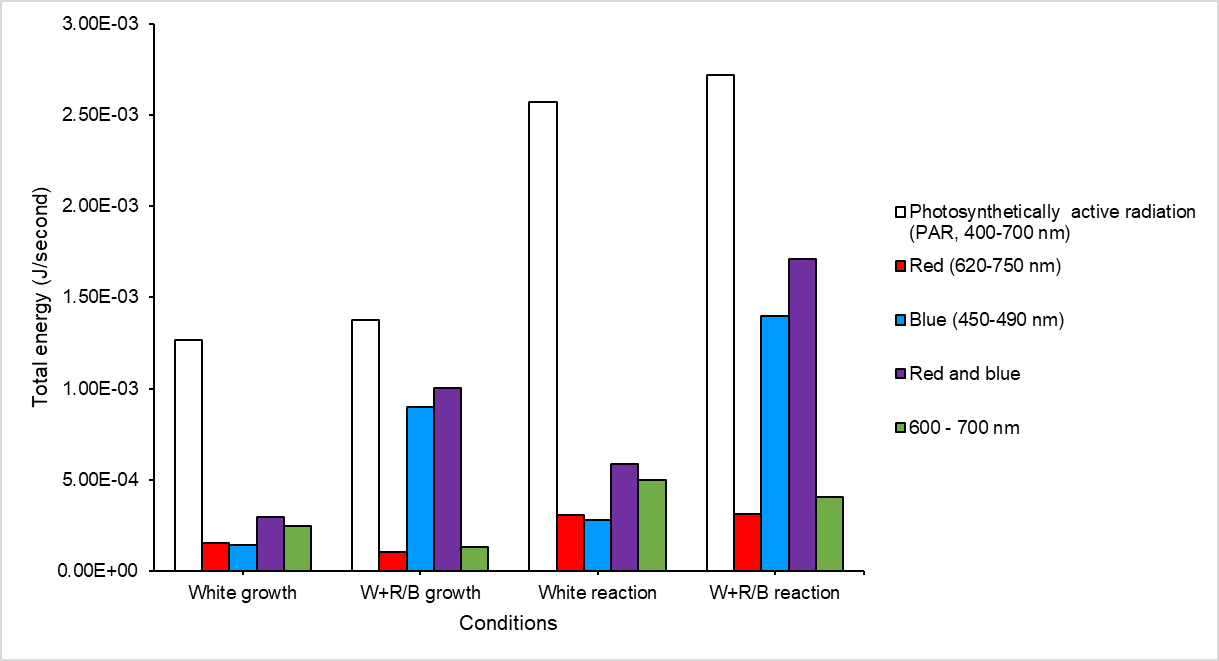


Figure S2. Light spectra comparison of White and W+R/B illumination and the total energy calculation at growth (150 µmol_photons_ m^‑2^ s^‑1^) and reaction (300 µmol_photons_ m^‑2^ s^‑1^) conditions. Spectra were measured using SpectaPen (PSI, Czechia).


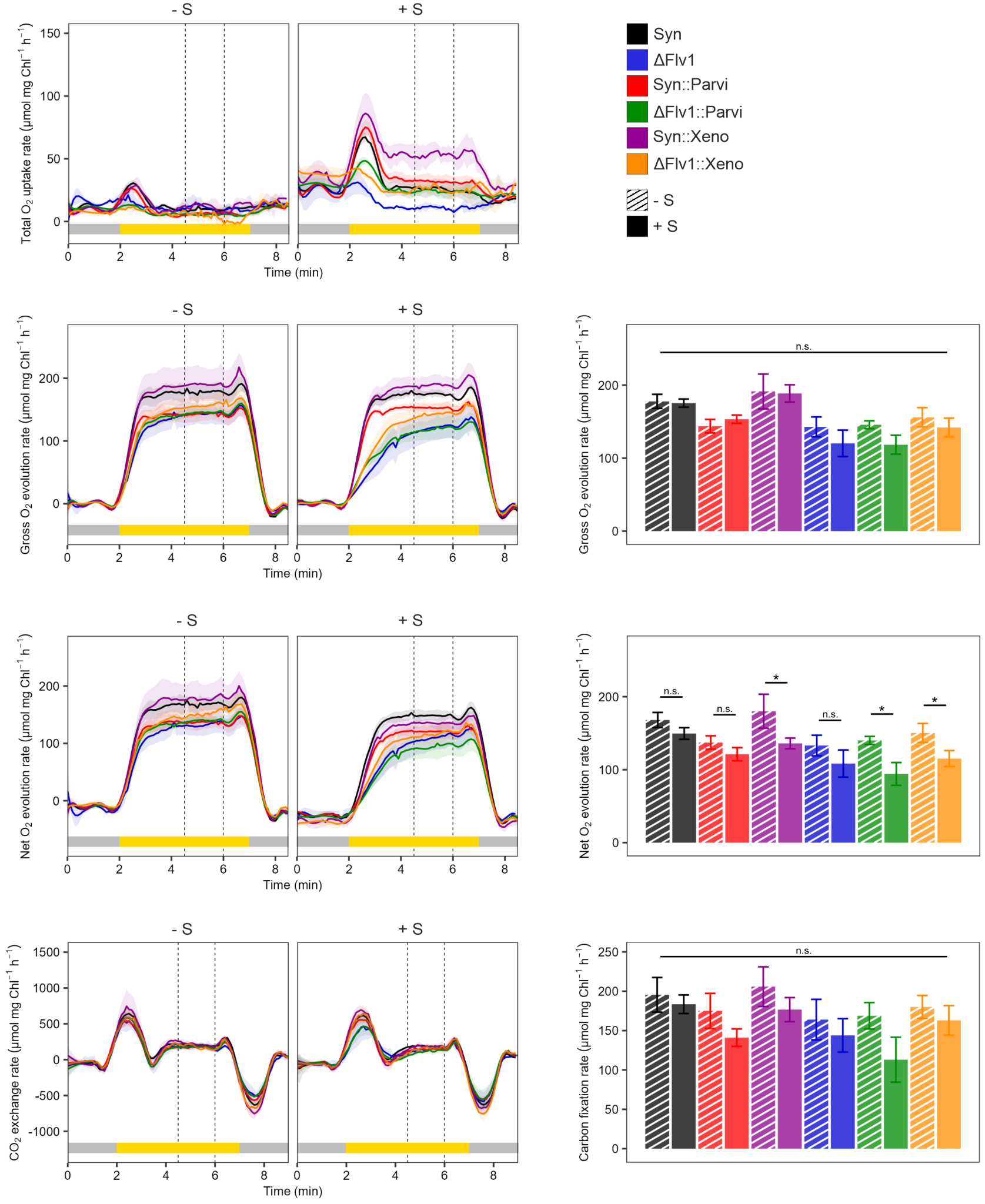


Figure S3. Gas exchange kinetics in the absence (- S) and presence (+ S) of cyclohexanone in Syn::Xeno and ΔFlv1::Xeno in LC conditions (0.04 % CO_2_). The column graphs represent Mean ± SD from the steady-state rate (4.5 - 6 min of experimental time). Black - Syn, blue - ΔFlv1, red - Syn::Parvi, green - ΔFlv1::Parvi, magenta - Syn::Xeno, orange - ΔFlv1::Xeno, striped - -S, full - +S. Statistical significance was tested by t-test, *≤0.05. P values can be found in Table S1.


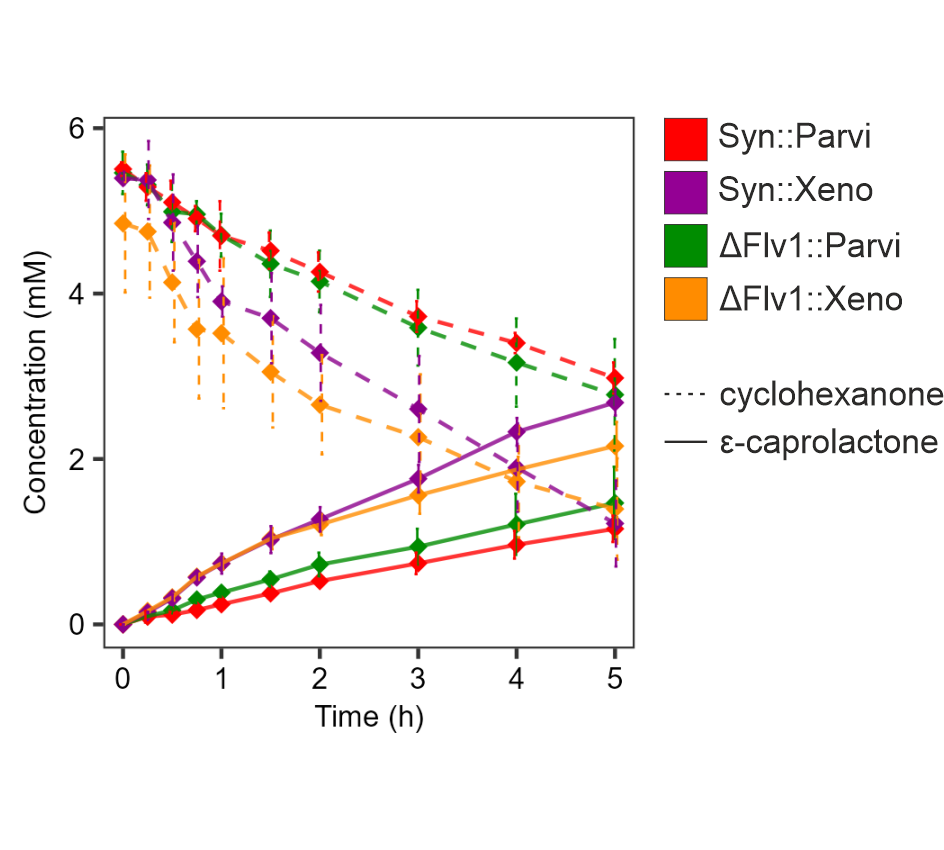


Figure S4. The concentration of cyclohexanone and ε-caprolactone over the course of 5 h in LC. Red - Syn::Parvi, green - ΔFlv1::Parvi, magenta - Syn::Xeno, orange - ΔFlv1::Xeno, dashed line - cyclohexanone, solid line - ε­caprolactone.


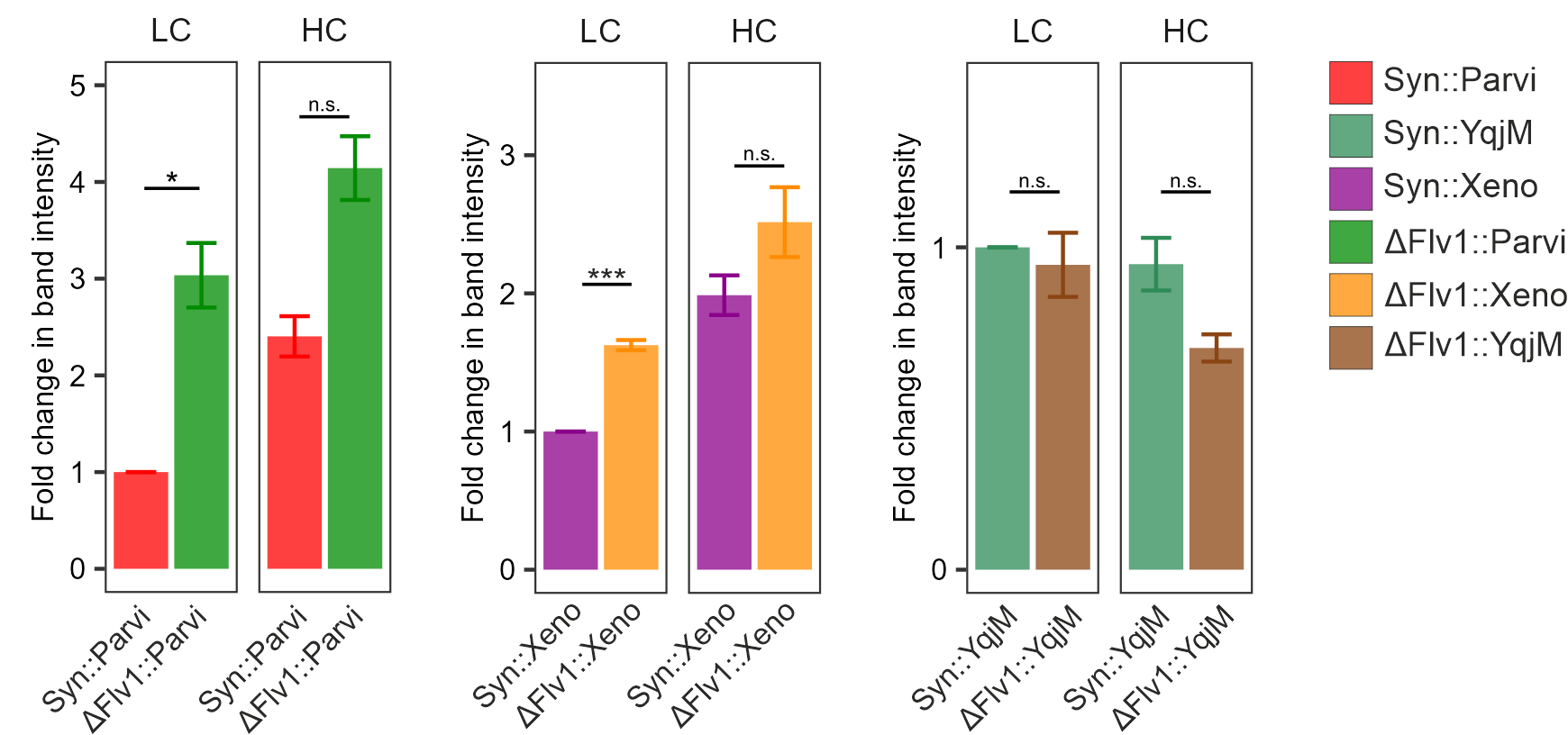


Figure S5. Relative protein abundance changes of Parvi, Xeno or YqjM in LC vs HC conditions. Samples were normalised to the Syn::Parvi, Syn::Xeno, or Syn::YqjM in LC conditions. The column bars represent Mean ± SEM. Red - Syn::Parvi, green - ΔFlv1::Parvi, magenta - Syn::Xeno, orange - ΔFlv1::Xeno, seagreen - Syn::YqjM, brown - ΔFlv1::YqjM. Statistical significance was tested by t-test, *≤0.05. P values can be found in Table S1.


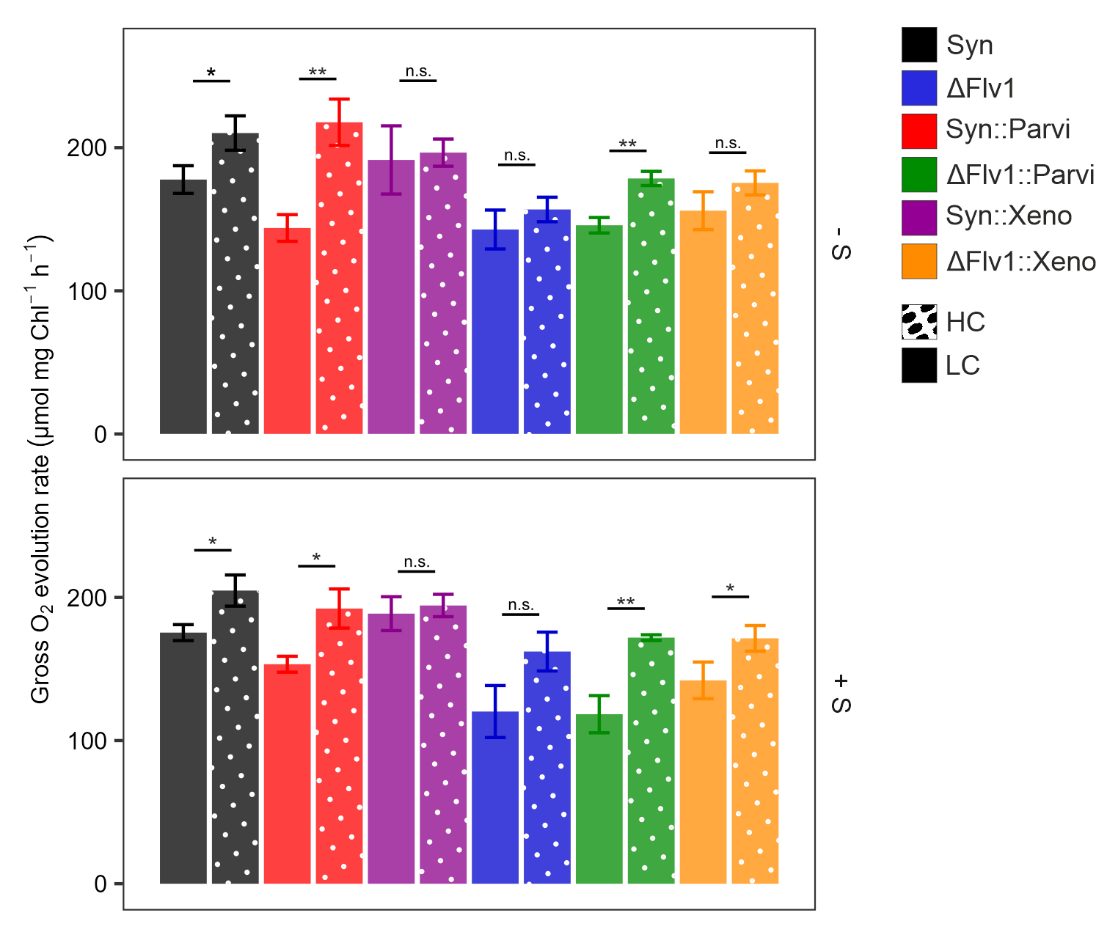


Figure S6. Gross O_2_ evolution in LC compared to HC conditions in the absence (- S) and presence (+ S) of cyclohexanone in Syn::Xeno and ΔFlv1::Xeno. The column graphs represent Mean ± SD from the steady-state rate (4.5 - 6 min of experimental time). Black - Syn, blue - ΔFlv1, red - Syn::Parvi, green - ΔFlv1::Parvi, magenta - Syn::Xeno, orange - ΔFlv1::Xeno, dotted – HC (3% CO_2_), full – LC (0.04% CO_2_). Statistical significance was tested by t-test, *≤0.05, **≤0.01. P values can be found in Table S1.


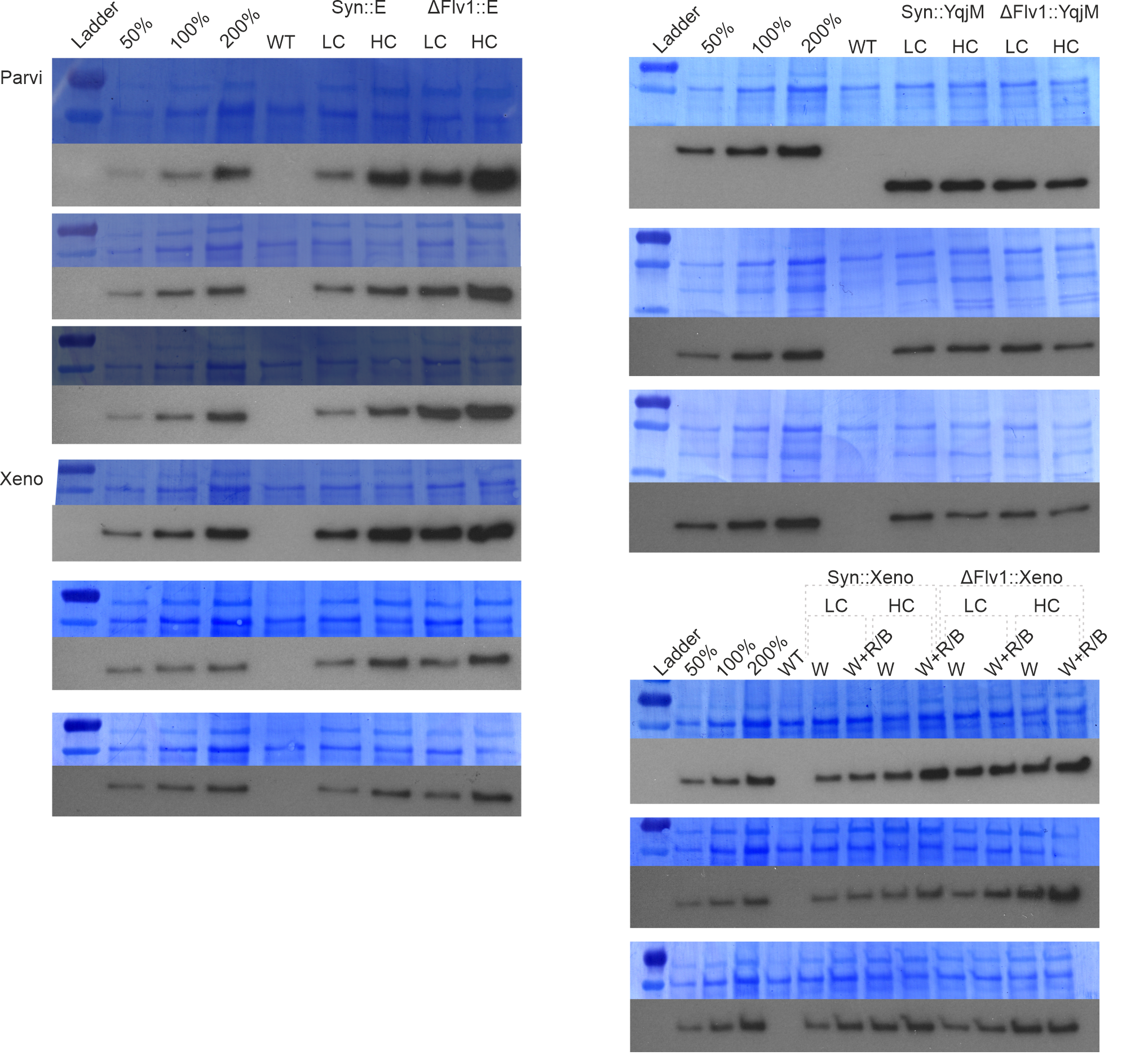


Figure S7. Immunodetection of BVMO enzymes and YqjM with an anti-His antibody. Syn::E - Synechocystis expressing Parvi or Xeno, ΔFlv1::E - ΔFlv1 strain expressing Parvi or Xeno, LC - 0.04% CO_2_, HC - 3% CO_2_, W - white light, W+R/B - white light enriched with red and blue wavelengths.


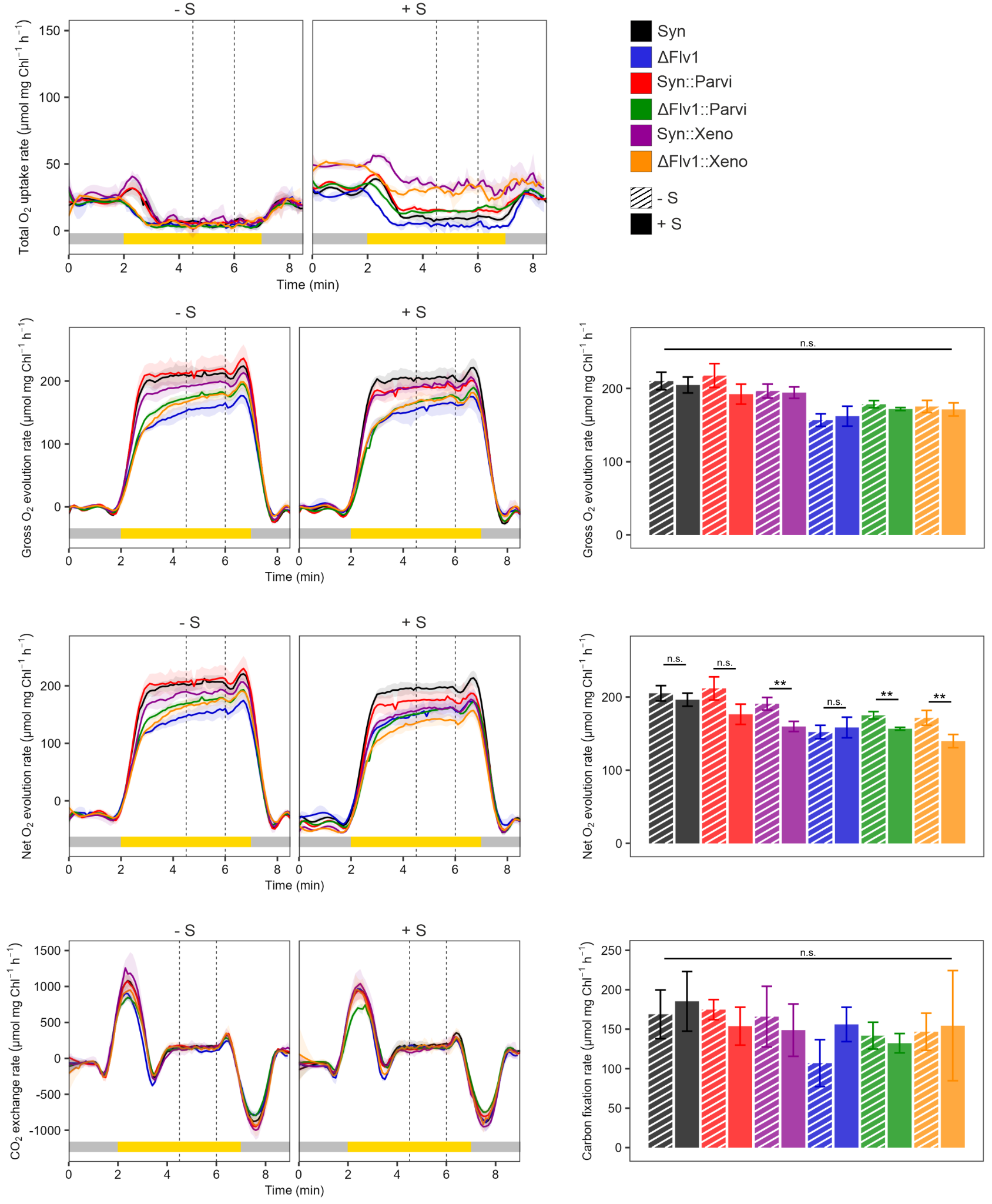


Figure S8. Gas exchange kinetics in the absence (- S) and presence (+ S) of cyclohexanone in Syn::Xeno and ΔFlv1::Xeno in HC conditions (3 % CO_2_). The column graphs represent Mean ± SD from the steady-state rate (4.5 - 6 min of experimental time). Black - Syn, blue - ΔFlv1, red - Syn::Parvi, green - ΔFlv1::Parvi, magenta - Syn::Xeno, orange - ΔFlv1::Xeno, striped - -S, full - +S. Statistical significance was tested by t-test, *≤0.05, **≤0.01. P values can be found in Table S1.

**Supplementary Tables**

Table S1. Results of statistical tests for main figures 1 - 4 and supplementary figures 3, 5, 6 and 8.


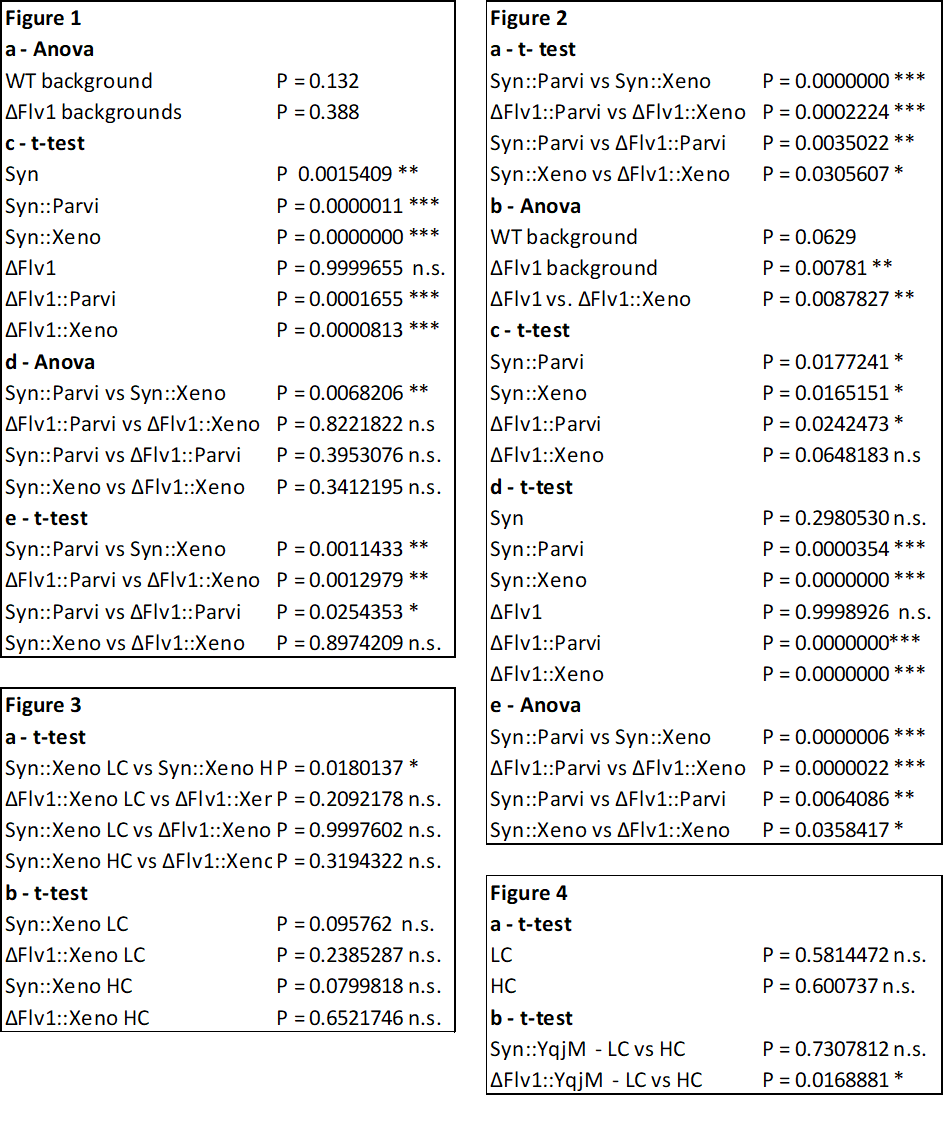

Supplement: Supplementary file 1 — Additional file 1. [file 12934_2025_2828_MOESM1_ESM.docx]
